# Supplementary material for: EIM: An effective solution for improving multi-modal large language models
Source: PLoS One. 2025 Aug 11;20(8):e0329590. doi: 10.1371/journal.pone.0329590 (PMC12338808; doi:10.1371/journal.pone.0329590)
Supplement: Attachment — Submitted filename: Response to Reviewers.pdf [file pone.0329590.s002.pdf]

EIM: An Effective Solution for Improving Multi-Modal Large Language Models

1 Additional Editor Comments:

This paper presented an EIM framework for enhanced multi-modal LLM training, focusing on the CLIP-ST-LLM architecture. The authors proposed 3 losses to improve the interaction among CLIP image/text encoder, ST network, and LLM, and designed a segmented training method to stabilize the joint training. They did experiments and analyzed the results. There are some points the authors should take care of as follows:

1. They should explain in detail why they only focused on the CLIP-ST-LLM architecture.

A: We add the explanation in the introduction Line 25-32. Following the three-segment structure, some recent works, like Emu3 [1], Show-o [2], InternVL3 [3], Janus-Pro [4], extend the multimodal LLM ability from visual understanding to visual understanding and generation by introducing the additional image decoder. However, the training costs of this new structure are too expensive. Its biggest highlight, image generation, is a bit lackluster because it can only generate an image that is the same as the model's input image, lacking practical application scenarios. Therefore, we only consider the three-segment structure in this paper.

2. Did they have any improvement on CLIP, ST, or LLM architecture?

A: No improvements to the three-segmented architecture. We focus on the losses instead of the architecture.

3. How to design three losses should be presented in detail.

A: We have presented a figure, a formula, and a detailed description of each loss. The reviewer's misunderstanding of our method may stem from the application of contrastive learning technology in the multimodal LLM field. We have added a description of contrastive learning in the Method section. Following the previous contrastive learning works [5–10], there is a queue $K = (k^1, \cdots, k^i, \cdots, k^N)$ to store key vectors for all samples, the positive sample refer to the input sample itself, and the negative samples refer to all other samples in the dataset. The details are shown in Fig 1. The contrastive loss aims to maximize the similarity between the query vector q^i and the key vector k^i, while minimizing the similarity between q^i and all other k^j, where $j \neq i$.

4. They should explain how they chose all parameters in their proposed model.

A: The temperature coefficient τ is a universal technique used to control the output distribution to be steeper or smoother. Following the previous works [6, 10], we set it to 0.07. We added the explanation in the Implementation details section.

5. They should do more experiments with another LLM in Coco dataset.

A: We added the reason why we only focused on the CLIP-ST-LLM architecture in the Introduction section. We do not intend to extend our solution to the new architecture multimodal LLM like Emu3 for more experiments right now.

6. They should use more metrics to evaluate the result.

A: We have presented all metrics that the dataset can provide. For the COCO caption, we have presented the BLEU4, METEOR, CIDEr, SPICE, and ROUGE_L metrics. For ScienceQA, we have presented the accuracy on each subtasks, and the

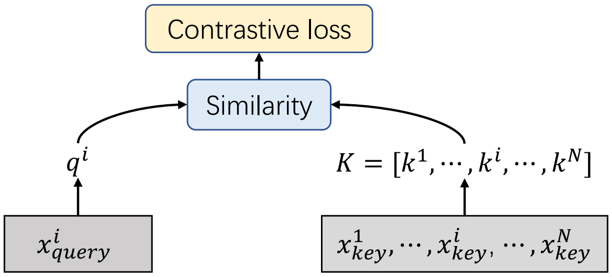

Fig 1. The overview of contrastive learning. For x^i_{query}, the positive sample is the x^i_{key}, the negative samples are x^j_{key}, where $j \neq i$. The larger the queue K, the richer the visual information and features it can represent. Then, when using queries for comparative learning, the more features of images can be learned. Therefore, previous works usually use all samples to build the queue K.

average accuracy. For MME, we have presented the scores on each subtasks, and the total scores.

7. In Table 1, GPT-2 is not equal with another metric, they should redesign the table.

A: We redesigned this table. The details are shown in Table 2 in the manuscript.

8. They should explain in detail why their proposed method got the best result.

A: We added a new Table 1 about the settings of segmented training, a new Table 5 to supplementary explanation of the effect of segmented training, and a new Fig 2 (Fig 7 in the manuscript) to present the examples.

9. The ablation study should be extended.

A: We added a new Table 1 about the settings of segmented training, a new Table 5 to supplementary explanation of the effect of segmented training, and a new Fig 2 (Fig 7 in the manuscript) to present the examples.

10. They should do more experiments, analyze the result in detail and compare with advanced methods in recent year.

A: We have applied our solution to the LaVIN and LLaMA-Adapter, which are the advanced multimodal LLMs in 2023 and 2024 years. We have explained in the introduction why we did not compare with other architecture models. The main reason is that these methods are concurrent with our work, and they require high-requirements of GPUs. We are unable to use RTX 3090 for training.

2 Reviewer #1 Comments:

The topic is new and useful, and improvements have been made to the Multi-Modal Large Language Models. The presentation is good, and the contributions are presented clearly. The research only needs examples or a case study.

A: We added a new Fig 2 to present the examples that are named Fig 7 in the manuscript.

3 Reviewer #2 Comments:

The paper addresses a timely and relevant challenge in enhancing multi-modal large language models by proposing a novel training approach that leverages contrastive losses across the image encoder, semantic transformation network, and language model. A key strength lies in its focus on improving performance without increasing model size significantly, highlighting parameter efficiency as a core contribution. The evaluation is

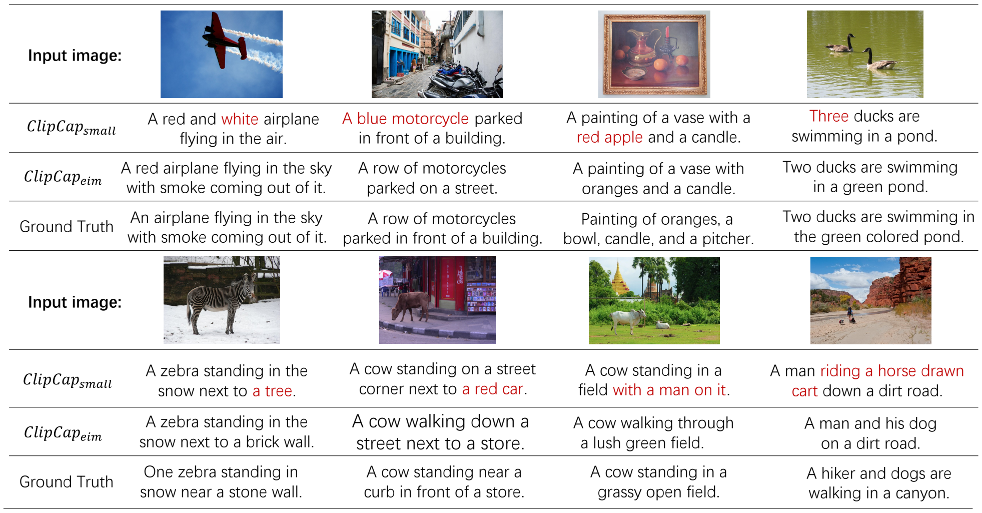

Fig 2. Case study of applying EIM to ClipCap$_{small}$ on the COCO Caption dataset. We show the caption generated by ClipCap$_{small}$, the caption generated by ClipCap$_{eim}$, and the ground truth. ClipCap$_{eim}$ generates more accurate and detailed captions than ClipCap$_{small}$. Incorrect text is highlighted in red.

comprehensive, spanning image captioning (COCO), multi-modal QA (ScienceQA), and benchmark tasks (MME), and the results consistently show measurable gains. Overall, the work offers a lightweight and practical strategy for boosting multi-modal model capabilities. However, there are certain areas which can benefit from improvements significantly.

1. While the paper is generally well-written, several methodological details are under-explained. In particular, the segmented training strategy needs clearer exposition. It is not fully explained how the training is segmented: for example, how many stages there are, what data is used in each stage, and how objectives change. A diagram or pseudocode would help. Also, the contrastive loss formulation is not precisely defined in the text – what are the positive/negative pairs, what temperature or distance metric is used, etc. These omissions make it hard to reproduce the work. The authors should ensure that Sections 3.1–3.2 clearly define all components (perhaps add an algorithm box or detailed equations for each objective).

A: We add a new subsection "Segmented training" in the Methods section, which includes a detailed explanation and a table. Contrastive learning is applied to approach positive samples and stay away from negative samples. We have added a description of contrastive learning in the Method section. Following the previous contrastive learning works [5–10], there is a queue $K = (k^1, \cdots, k^i, \cdots, k^N)$ to store key vectors for all samples, the positive sample refer to the input sample itself, and the negative samples refer to all other samples in the dataset. The contrastive loss aims to maximize the similarity between the query vector q^i and the key vector k^i, while minimizing the similarity between q^i and all other k^j, where $j \neq i$. The details are shown in Fig 1.

2: The related works or background literature needs to be enhanced. Related literature on parameter-efficient adaptation is thin. The claim of "parameter efficiency" warrants discussion of known techniques. For example, adapter module and LoRA enable fine-tuning with very few parameters. BLIP-2 also reports massive savings. The authors should cite such methods to frame their efficiency claim. Additionally, recent surveys of multimodal LLMs or vision-language models could provide context (e.g. recent surveys on multimodal LLMs). If possible, explicitly compare EIM's added parameter count to these baselines. Without this, the efficiency claim is ungrounded.

Include citations and discussion of relevant multimodal models. Compare EIM conceptually to other methods. This will help readers gauge novelty and novelty of approach.

A: Firstly, our work focuses on losses. Unlike the parameter-efficient methods, our method does not modify the architecture of the model. Secondly, we claim that our solution is effective instead of parameter efficiency. Our solution can help CLipCap$_{small}$ achieves better performance than CLipCap$_{large}$, and CLipCap$_{large}$ is 3.13 times the number of CLipCap$_{small}$. Then, we have added more PETL(Parameter-Efficient Transfer Learning) applied models [11, 12, 12–23] in Line 109 in related work section. Finally, we added the parameter count in Table 2 in the manuscript.

3. The results are encouraging but could be strengthened by additional analysis. Just a suggestion to include an experiment, which I agree, might be difficult at this stage. For instance, a study isolating the contribution of the contrastive loss is critical: does removing the contrastive term degrade performance? Similarly, what is the effect of the segmentation (e.g. training all objectives at once versus in phases)?

A: In our manuscript, Table 3 presents the results of the proposed losses applied individually, and Table 4 presents the effect of the segmentation. To present the effect of segmented training more intuitively, we added a new Table 5 in the manuscript. When we use $loss_{base}$, $loss_{ST}$, $loss_{LM}$, and $loss_{IE}$ together in one training stage, the performance is 33.0%. When we use segmented training, the performance is improved to 33.9% with a 0.9% improvement.

4. Also, the evaluation metrics should be clearly reported (e.g. BLEU/CIDEr for COCO captions, accuracy for QA), and error bars or statistical significance mentioned if possible. Report standard evaluation metrics with baselines (include performance of a plain LLM or existing vision-language model for each task). For COCO Captions, provide quantitative scores and maybe example captions. For ScienceQA and MME, clearly list accuracy gains. If possible, include comparisons to known results (e.g. "EIM achieves X%, compared to Y% from [baseline]").

A: The evaluation metrics are reported in the Datasets and Metrics section. The error bars can not be presented due to the high cost of training multimodal LLM. We add the improvement gains in Tables 2-5, Table 10, and Table 11 in the manuscript.

5. The manuscript should temper broad claims until supported by evidence. For example, stating "EIM reduces trainable parameters by X%" requires precise accounting: list the total parameters of the base model, parameters tuned or added by EIM, and how this compares to full fine-tuning. Works like LoRA demonstrate drastic parameter reduction (10,000x fewer trainable parameters on GPT-3). If EIM's savings are more modest, the language should reflect that. Similarly, any claim of achieving "state-of-the-art" should specify baselines. If claiming SOTA on ScienceQA or MME, the paper should compare numerically to the best published numbers (e.g. LLaVA's 92.5% on ScienceQA). Otherwise, phrasing like "improves over baseline" is safer.

A: We added the parameter number in Table 2 in the manuscript. The numbers are precise. We have not claimed the state-of-the-art in this paper.

4 Reviewer #3 Comments:

In this paper, the authors proposed an EIM framework for enhanced multi-modal LLM training, focusing on the CLIP-ST-LLM architecture. Compared with traditional base loss design, the authors proposed 3 new contrastive learning losses to improve the interaction among CLIP image/text encoder, ST network, and LLM, and designed a segmented training method to stabilize the joint training. The results on multiple open sourced data sets show that all 3 losses designed improve the vision LLM's captioning and reasoning ability, and the models trained based on the proposed methods achieved

better performance than the baseline models with more parameters. Despite the strength mentioned above, I have the following concerns.

1. General applicability. The authors designed and tested the proposed methods for CLIP-based vision LLMs. While ClipCap, LaVIN and Llama-adapter are representative for CLIP-based models, there are many state-of-the-art vision models not using CLIP or CLIP-like encoders at all, like Emu3 [1]. When CLIP is not applied, are the proposed losses still useful? The authors should consider adding results for models not using CLIP where only part of the 3 proposed losses are applicable, and discussing the general applicability of the proposed losses on a wider range of architectures.

A: Firstly, Emu3 trains the vision tokenizer based on SBER-MoVQGAN5, which can encode a 4 × 512 × 512 video clip or a 512 × 512 image into 4096 discrete tokens from a codebook of size 32,768. This means CLIP is also applied in Emu3. Models like Emu3 [1], Show-o [2], InternVL3 [3], Janus-Pro [4], extend the ability from visual understanding to visual understanding and generation by introducing an additional image decoder in the traditional CLIP-ST-LLM architecture. Secondly, works like Emu3, Show-o, internVL-3, Janus-Pro are concurrent with our work, and these works require high-requirements of GPUs. We are unable to use RTX 3090 for training. Finally, we only focus on the three-segmentation architecture right now and we added the explanation in the introduction Line 25-32.

2. Notations. In Eq. (2)(3)(4), the losses authors defined seem to be the loss for one sample i, rather than the whole data set. The authors should consider using $loss_{IE}(i)$ rather than $loss_{IE}$, or express the loss of the whole data set, to make the expression more clear.

A: We modified the Eq. (2)(3)(4).

3. Batch size for contrast learning. Under Eq. (2)(3)(4), the authors defined N as the number of the pair samples in the whole training set, while in contrastive learning, typically a small batch like 5 is used for contrast to guarantee a feasible computing and benefit from stochasticity, rather than using the whole training set. The authors should clarify why the batch for contrast is so large.

A: Following the previous contrastive learning works [5–10], there is a queue $K = (k^1, \cdots, k^i, \cdots, k^N)$ to store key vectors for all samples, the positive sample refer to the input sample itself, and the negative samples refer to all other samples in the dataset. In general, the larger the queue K, the richer the visual information and features it can represent. This way, when using queries for comparative learning, the more features of images can be learned. Thus, previous works usually use all samples to build the queue K. The details are shown in Fig 1.

4. Setting of tau. The softmax temperature tau is set in all 3 losses with the same default value 0.07, without more explanations or results in the later sections. How does this hyperparameter influence the training performance? The authors should consider adding a figure to show how different tau settings influence the results.

A: The temperature coefficient τ is a universal technique used to control the output distribution to be steeper or smoother. Following the previous works [6, 10], we set it to 0.07. We added the explanation in the Implementation details section.

5. Segmented training. In the Results section, the authors use a segmented training manner to minimize the 3 losses, instead of training the whole network jointly. As this method is essential to guarantee a stable convergence, the authors should consider moving it to the Methods section, and stating its motivation and details in a more systematic way.

A: We added a new subsection called “Segmented training” in the Method section.

6. Error bars. In result tables, the performance difference between using different segmented training orders is often small. The authors should consider reporting the performance standard deviation over multiple runs for the result tables, to make the

results and trends more convincing and reproducible.

A: Multimodal LLM papers, like LLaVA, LLaVA-1.5, BLIP-2, LLaMA-Adapter, and LaVIN, do not present the error bars. We speculate that this is because of the following two reasons. Firstly, for multimodal LLM, there is no dropout configuration during training. Secondly, the training costs of multimodal LLM are expensive.

7. Writing. Please fix the typos in the manuscript. For example, there is no GTX 3090, which should be RTX 3090. In Abstract, “researchs” → research.

A: We modified all ”GTX 3090” in this paper to ”RTX 3090” and the “researchs” in the abstract to ”research”.

References

1. Wang X, Zhang X, Luo Z, Sun Q, Cui Y, Wang J, et al. Emu3: Next-Token Prediction is All You Need; 2024. Available from: https://arxiv.org/abs/2409.18869.
2. Xie J, Mao W, Bai Z, Zhang DJ, Wang W, Lin KQ, et al. Show-o: One Single Transformer to Unify Multimodal Understanding and Generation; 2024. Available from: https://arxiv.org/abs/2408.12528.
3. Zhu J, Wang W, Chen Z, Liu Z, Ye S, Gu L, et al. InternVL3: Exploring Advanced Training and Test-Time Recipes for Open-Source Multimodal Models; 2025. Available from: https://arxiv.org/abs/2504.10479.
4. Chen X, Wu Z, Liu X, Pan Z, Liu W, Xie Z, et al. Janus-Pro: Unified Multimodal Understanding and Generation with Data and Model Scaling; 2025. Available from: https://arxiv.org/abs/2501.17811.
5. Mokady R, Hertz A, Bermano AH. Clipcap: Clip prefix for image captioning. arXiv preprint arXiv:211109734. 2021;.
6. He K, Fan H, Wu Y, Xie S, Girshick R. Momentum contrast for unsupervised visual representation learning. In: Proceedings of the IEEE/CVF conference on computer vision and pattern recognition; 2020. p. 9729–9738.
7. Chen X, Fan H, Girshick R, He K. Improved baselines with momentum contrastive learning. arXiv preprint arXiv:200304297. 2020;.
8. Chen T, Kornblith S, Norouzi M, Hinton G. A simple framework for contrastive learning of visual representations. In: International conference on machine learning. PmLR; 2020. p. 1597–1607.
9. Chen X, Xie S, He K. An empirical study of training self-supervised vision transformers. In: Proceedings of the IEEE/CVF international conference on computer vision; 2021. p. 9640–9649.
10. Wu Z, Xiong Y, Yu SX, Lin D. Unsupervised feature learning via non-parametric instance discrimination. In: Proceedings of the IEEE conference on computer vision and pattern recognition; 2018. p. 3733–3742.
11. Houlsby N, Giurgiu A, Jastrzebski S, Morrone B, De Laroussilhe Q, Gesmundo A, et al. Parameter-efficient transfer learning for NLP. In: International conference on machine learning. PMLR; 2019. p. 2790–2799.
12. Luo G, Huang M, Zhou Y, Sun X, Jiang G, Wang Z, et al. Towards Efficient Visual Adaption via Structural Re-parameterization. arXiv preprint arXiv:230208106. 2023;.

13. Li XL, Liang P. Prefix-Tuning: Optimizing Continuous Prompts for Generation. In: Zong C, Xia F, Li W, Navigli R, editors. Proceedings of the 59th Annual Meeting of the Association for Computational Linguistics and the 11th International Joint Conference on Natural Language Processing (Volume 1: Long Papers). Online: Association for Computational Linguistics; 2021. p. 4582–4597.

14. Zhang R, Han J, Liu C, Zhou A, Lu P, Li H, et al. LLaMA-Adapter: Efficient Fine-tuning of Large Language Models with Zero-initialized Attention. In: The Twelfth International Conference on Learning Representations; 2024.

15. Jia M, Tang L, Chen BC, Cardie C, Belongie S, Hariharan B, et al. Visual Prompt Tuning. In: European Conference on Computer Vision (ECCV); 2022.

16. Hu EJ, Shen Y, Wallis P, Allen-Zhu Z, Li Y, Wang S, et al. LoRA: Low-Rank Adaptation of Large Language Models. In: International Conference on Learning Representations; 2022.

17. Dettmers T, Pagnoni A, Holtzman A, Zettlemoyer L. QLoRA: Efficient Finetuning of Quantized LLMs. arXiv preprint arXiv:230514314. 2023;.

18. Gao P, Han J, Zhang R, Lin Z, Geng S, Zhou A, et al. LLaMA-Adapter V2: Parameter-Efficient Visual Instruction Model. arXiv preprint arXiv:230415010. 2023;.

19. Luo G, Zhou Y, Ren T, Chen S, Sun X, Ji R. Cheap and Quick: Efficient Vision-Language Instruction Tuning for Large Language Models. Advances in neural information processing systems (NeurIPS). 2023;.

20. Sung YL, Cho J, Bansal M. Vl-adapter: Parameter-efficient transfer learning for vision-and-language tasks. In: Proceedings of the IEEE/CVF conference on computer vision and pattern recognition; 2022. p. 5227–5237.

21. Zhang Y, Zhou K, Liu Z. Neural prompt search. IEEE Transactions on Pattern Analysis and Machine Intelligence. 2024;.

22. Liu X, Zheng Y, Du Z, Ding M, Qian Y, Yang Z, et al. GPT understands, too. AI Open. 2024;5:208–215.

23. Lester B, Al-Rfou R, Constant N. The Power of Scale for Parameter-Efficient Prompt Tuning. In: Proceedings of the 2021 Conference on Empirical Methods in Natural Language Processing; 2021. p. 3045–3059.
