# Supplementary material for: EIM: An effective solution for improving multi-modal large language models
Source: PLoS One. 2025 Aug 11;20(8):e0329590. doi: 10.1371/journal.pone.0329590 (PMC12338808; doi:10.1371/journal.pone.0329590)
Supplement: Attachment — Submitted filename: Review - PONE-D-25-16901.docx [file pone.0329590.s001.docx]

The paper addresses a timely and relevant challenge in enhancing multi-modal large language models by proposing a novel training approach that leverages contrastive losses across the image encoder, semantic transformation network, and language model. A key strength lies in its focus on improving performance without increasing model size significantly, highlighting parameter efficiency as a core contribution. The evaluation is comprehensive, spanning image captioning (COCO), multi-modal QA (ScienceQA), and benchmark tasks (MME), and the results consistently show measurable gains. Overall, the work offers a lightweight and practical strategy for boosting multi-modal model capabilities.

However, there are certain areas which can benefit from improvements significantly.

- While the paper is generally well-written, several methodological details are under-explained. In particular, the **segmented training strategy** needs clearer exposition. It is not fully explained how the training is segmented: for example, how many stages there are, what data is used in each stage, and how objectives change. A diagram or pseudocode would help. Also, the **contrastive loss** formulation is not precisely defined in the text – what are the positive/negative pairs, what temperature or distance metric is used, etc. These omissions make it hard to reproduce the work. The authors should ensure that Sections 3.1–3.2 clearly define all components (perhaps add an algorithm box or detailed equations for each objective).
- The related works or background literature needs to be enhanced. Related literature on parameter-efficient adaptation is thin. The claim of “parameter efficiency” warrants discussion of known techniques. For example, adapter module and LoRA enable fine-tuning with very few parameters. BLIP-2 also reports massive savings. The authors should cite such methods to frame their efficiency claim. Additionally, recent surveys of multimodal LLMs or vision-language models could provide context (e.g. recent surveys on MLLMs). If possible, explicitly compare EIM’s added parameter count to these baselines. Without this, the efficiency claim is ungrounded. Include citations and discussion of relevant multimodal models. Compare EIM conceptually to other methods. This will help readers gauge novelty and novelty of approach.
- The results are encouraging but could be strengthened by additional analysis. Just a suggestion to include an experiment, which I agree, might be difficult at this stage. For instance, a **study** isolating the contribution of the contrastive loss is critical: does removing the contrastive term degrade performance? Similarly, what is the effect of the segmentation (e.g. training all objectives at once versus in phases)?
- Also, the evaluation metrics should be clearly reported (e.g. BLEU/CIDEr for COCO captions, accuracy for QA), and error bars or statistical significance mentioned if possible. Report standard evaluation metrics with baselines (include performance of a plain LLM or existing vision-language model for each task). For COCO Captions, provide quantitative scores and maybe example captions. For ScienceQA and MME, clearly list accuracy gains. If possible, include comparisons to known results (e.g. “EIM achieves X%, compared to Y% from [baseline]”).
- The manuscript should temper broad claims until supported by evidence. For example, stating “EIM reduces trainable parameters by X%” requires precise accounting: list the total parameters of the base model, parameters tuned or added by EIM, and how this compares to full fine-tuning. Works like LoRA demonstrate drastic parameter reduction (10,000× fewer trainable parameters on GPT-3). If EIM’s savings are more modest, the language should reflect that. Similarly, any claim of achieving “state-of-the-art” should specify baselines. If claiming SOTA on ScienceQA or MME, the paper should compare numerically to the best published numbers (e.g. LLaVA’s 92.5% on ScienceQA). Otherwise, phrasing like “improves over baseline” is safer.

Based on the current state of the paper, I recommend a major revision.

While the core idea is promising and the experiments are compelling, the paper requires clearer methodological exposition, stronger positioning against prior work, more rigorous ablations, and precise articulation of its efficiency claims. These are essential to properly validate and contextualize the contribution before it can be accepted for publication.
